# Low cost and easy acquisition: corn grain in microsurgery training

**DOI:** 10.1590/0100-6991e-20223217-en

**Published:** 2022-12-09

**Authors:** MANUELA RODRIGUES NEIVA FERNANDES, DANIELA FERREIRA TRAMONTIN, ANTÔNIO LEONARDO JATAHI CAVALCANTI PIMENTEL, LUÍS VINÍCIUS PIRES DA COSTA, DÁRIO SANTANA, DÉBORA PINHEIRO XAVIER, LÍVIA GUERREIRO DE BARROS BENTES, DEIVID RAMOS DOS SANTOS

**Affiliations:** 1 - Universidade do Estado do Pará, Laboratório de Cirurgia Experimental - Belém - PA - Brasil

**Keywords:** Microsurgery, Simulation Training, Experimental Development, Suture Techniques, Microcirurgia, Treinamento por Simulação, Desenvolvimento Experimental, Técnicas de Sutura

## Abstract

**Objectives:**

develop an easily accessible model for training the initial motor practice in microsurgery using corn kernels.

**Methods:**

ten corn kernels (Zea mays) were used. A 7mm longitudinal cut was made on one side of the corn grain. The training consisted of performing 4 simple knots between the edges of the incision, using 10-0 mononylon thread. The parameters analyzed were 1) cost of the model; 2) assembly time of the model test system; 3) time for performing the knots; 4) distance between the knots.

**Results:**

in all corn kernels tested, it was possible to perform the proposed microsurgical suture training, without any difficulty in the procedure. The average time to perform the 4 knots was 6.51±1.18 minutes. The total cost of the simulator model was R$3.59. The average distance between the knots was 1.7±0.3mm. The model developed from corn grains has an extremely low cost when compared to the use of animals or high-tech simulators. Other advantages are the easy availability of canned corn kernels and the possibility of making more than four knots along the 7mm incision.

**Conclusion:**

the training model developed has low cost, is easy to acquire and viable for training basic manual skills in microsurgery.

## RESUMO

**Objetivo:**

desenvolver um modelo facilmente acessível para o treinamento da prática motora inicial em microcirurgia a partir da utilização de grãos de milho.

**Métodos:**

foram utilizados dez grãos de milho (Zea mays). Realizou-se um corte longitudinal de 7mm em uma das faces do grão de milho. O treinamento consistiu na realização de 4 pontos simples entre as bordas da incisão, utilizando fio de mononáilon 10-0. Os parâmetros analisados foram 1) custo do modelo; 2) tempo de montagem do sistema de teste do modelo; 3) tempo de realização dos nós; 4) distância entre os pontos.

**Resultados:**

em todos os grãos testados foi possível realizar o treinamento de sutura microcirúrgica proposto, sem dificuldade ao procedimento. O tempo médio para a realização dos 4 pontos foi de 6,51±1,18 minutos. O custo total do modelo simulatório foi de R$3,59. A distância média entre os pontos foi de 1,7±0,3mm. O modelo desenvolvido a partir de grãos de milhos apresenta custo extremamente baixo quando comparado ao uso de animais ou de simuladores de alta tecnologia. Outras vantagens são fácil disponibilidade de grãos de milho em conserva e possibilidade de serem realizados mais de quatro pontos ao longo da incisão de 7mm.

**Conclusão:**

o modelo de treinamento desenvolvido é de baixo custo, de fácil aquisição e viável para o treinamento de habilidades manuais básicas em microcirurgia.

## INTRODUCTION

In the traditional learning model in surgery “see one, do one, teach one”, established by Halsted^1^, skills are taught to medical residents and students during the surgical procedure^2^. However, this system is going into obsolescence due to the importance of ensuring patient safety, which guarantees the development of skills by young surgeons, characteristics that cannot be obtained only by mere observation, but especially by repetition training^3^ ^,^ ^4^.

Faced with a more complex surgical perspective, the learning curve for competence in microsurgery involves, in addition to high technical preparation, skillful decision-making and correct time management, which broadens the focus on simulation, so that live animal models, especially rats, are the most used^4^ ^,^ ^5^.

However, the use of biological materials is regulated by international and national standards of experimentation, with handling demanding complex and costly logistics for institutions and students, by requiring, for example, the use of anesthetics, approval by the review board, and guarantee of a place to protect the animals^6^. Furthermore, the ethical establishment of the process of using in vivo models for educational purposes involves following the principle of the 3Rs, “refinement, replacement, and reduction”, and the active search for the reduction in the use of animals^7^ ^,^ ^8^.

Thus, non-living microsurgical simulation models have been an alternative, since they tend to be easy to store, low cost, non-infectious and simple to assemble^4^ ^,^ ^9^. Therefore, given the possibility of non-living materials such as cereals being used in the first steps of microsurgical learning^10^, this study aims to develop an easily accessible model for training initial motor practice in microsurgery using corn grains.

## METHODS

This research is characterized as an experimental and cross-sectional study, carried out at the Laboratory of Experimental Surgery at the Pará State University. The Brazilian laws on the breeding and use of animals were followed (Law Nº11,794 /08).

For the training model, we used corn grains (Zea mays), preserved in water and salt, purchased at a local supermarket and kept in an airy environment until the beginning of the study. Initially, the contents of the can were filtered, and the water and salt drained. Ten units of corn grains were used, and the procedures were performed in sequence. Each grain was dried with paper towels and fixed on a surgical board with the aid of a tape, which was removed for the photos to be taken. Then, under the videomagnification system, a 7mm longitudinal cut was made on one side of the corn grain.

The magnification system^11^ ^,^ ^12^ that enabled the capture of images in this study contains a Sony^©^ Handycam HDR-XR160 camera connected to a 55’ Curved Full HD TV via an HDMI cable. Two fluorescent light sources were used next to the board to provide adequate illumination of the operative field. The training consisted of performing 4 simple sutures between the edges of the incision, using 10-0 mononylon thread with an 80µm needle (3mm in length and 3/8 of a circle). The distance between the points was measured with the aid of graph paper, and the time was recorded using a digital stopwatch handled by an assistant. The procedure was performed by a surgeon with more than 3 years of experience in microsurgery. Performance was evaluated by a microsurgeon with more than 15 years of experience and classified according to a scale adapted from Santos^13^ (Chart 1).

**Chart 1:** Performance rating scale.

Domain	Score		
	1	2	3
1. Tissue handling	Frequently used unnecessary force on tissue or caused tissue damage	Carefully manipulated tissue, but occasionally caused inadvertent damage	Consistently manipulated tissue appropriately, causing minimal damage
2. Handling of instruments	Constantly makes hesitant or awkward movements with instruments	Competent use of instruments, although occasionally sluggish or clumsy	Tuned and fluid movements with the instruments
3. Movements	Too many unnecessary moves	Efficient moves, but some unnecessary	Evident economy of movement and maximum efficiency
4. Ergonomics	Improper positioning that makes it difficult to perform the procedure	Improper positioning that can make it difficult to perform the procedure	Positions perfectly in the operative field
5. Tremors	Presence of macroscopic tremors	Tremors that do not affect procedure performance	Absence of fine tremors
6. Suturing technique	Clumsy and insecure, tying knots improperly and unable to maintain tension	Careful and slow, with most knots properly placed with proper tension	Excellent suture control with proper knot placement and correct tension
Domain	Score		
	1	2	3
7. Operation flow	Often hesitated in performing the procedure and appeared unsure	Demonstrated some planning for performing the procedure, with reasonable progression of steps	Operation performed efficiently, with proper progression from one movement to another

**Figure 1:**
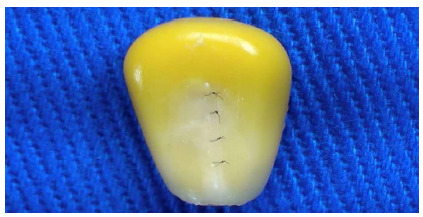
Corn grain suture under videomicromagnification.

**Figure 2:**
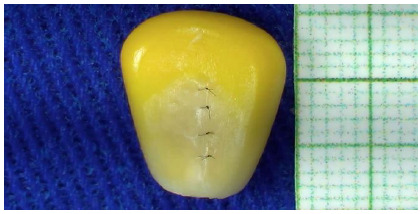
Corn grain suture under videomicromagnification.

The analyzed parameters were 1) cost of the model, 2) model test system assembly time, 3) duration of suturing, 4) surgeon performance, and 5) average distance between stitches. Microsoft Word and Excel® software were used for data analysis, graphics creation, and photo editing.

We used the BioEstat^©^ 5.4 software for statistical analysis. We evaluated the Pearson’s correlation coefficient based on the time required for surgery and the order of procedures. We adopted a significance level of 5%.

## RESULTS

In all corn grains tested, it was possible to perform the proposed microsurgical suture training, without difficulty in the procedure, guaranteeing the viability of the simulation model. Regarding the viability of making the suture, it is due to the longitudinal arrangement of the fibers, which prevents sliding and facilitates the tightening of the knots, allowing the correct surgical technique.

Assembly of the training system took about 4.54 minutes. Faced with an average distance between the stitches of 1.7±0.3mm, the average time to perform the 4 stitches was 6.51±1.18 minutes, the first training taking 8.39 minutes, and the last, 4.58 minutes. The total cost of the simulative model was R$3.59, relative to a package of 280g of canned corn. Correlation analysis between time and order of procedures showed a reduction in the time required to perform surgery (Pearson rho: -0.39, 95% C: -0.26/-0.90, p<0.5). The surgeon’s mean score on the performance scale was 19.7±1.1 points (Chart 1).

## DISCUSSION

Faced with a context of continuous efforts to improve the quality of microsurgical training and to reduce dependence on animal models, alternative simulative materials are relevantly useful in the progression of the motor technique of undergraduates and residents^14^ ^,^ ^15^. In this study, an easy-to-build, low-cost model was developed for initial manual training in microsurgical suturing.

The use of delicate skills in microsurgery covers several surgical specialties, having as a complexity factor an extensive learning curve, as it requires the development of precision techniques and fine manipulation^13^ ^,^ ^16^. With regard to the acquisition of motor precision, it is streamlined by the use of microsurgical simulation mechanisms, which allow greater independence and safety in learning, without imposing risks to the lives of patients^16^. Furthermore, the use of simulation models allows the development of specific training protocols suitable for the level of undergraduates and residents^17^. This perspective, when associated with the use of non-living simulation materials, also has the benefit of reducing the use of animals, promoting the acquisition of satisfactory skills to perform procedures and the optimization of microsurgical practice^13^.

In the current perspective of the practice of skills in microsurgery, expenses related to reality simulator devices are the most impactful for laboratories^18^. As for the devices used in training, the videomagnification system corresponds to an obstacle commonly subject to attenuation, especially in view of the practice of residents, who may have surgical instruments and microscopes in their teaching hospitals^11^ ^,^ ^17^, and the possibility of adaptations, such as the use of smartphones^19^ or tablets^20^ for magnification in training. The simulation model developed from corn grains has an extremely low cost when compared with the use of animals or high-tech simulators, which acquires even greater relevance in view of the usual financial limitations of microsurgery training laboratories in developing countries, such as Brazil^17^ ^,^ ^20^. When compared with other non-animal models, corn is found to be more expensive than tomatoes, but less expensive than grapes, both developed for the practice of ophthalmic microsurgery^2^ ^,^ ^13^.

Among the parameters that can be evaluated in this study, the time for making the suture consolidates the usefulness of the proposed model in the progression of fine motor skills. This is due to experience and the sequence of practices that provide skills gain and optimization of procedure time. It may be useful, therefore, as initial training for inexperienced residents before training on realistic models after reaching a certain degree of microsurgical precision^21^ ^-^ ^23^. Still, other advantages of the developed model are the easy availability of canned corn grains and the possibility of performing more than four stitches along the 7mm incision.

Quantitative (time for stitches) and qualitative (suture quality and surgeon’s performance) assessment are essential parameters to be studied. Such variables, when submitted to the learning curve, make training stimulating and challenging as there is a perception of the evolution of technical skills over time^17^ ^,^ ^20^, especially if this is done through a structured assessment^23^, as in the adaptation (Chart 1) of the ratings scale by Santos^13^.

Among the obstacles inherent to the developed model, there is the low fidelity to human anatomical structures, particularly associated with the arrangement of grain fibers, whose orientation makes the suture less resistant to traction than animal tissues carriers of collagen^24^. However, this limitation does not restrict the use of the model developed in the training of microsurgical skills when taking into account, for example, the proper manipulation of instruments, distance of the stitches in relation to the surgical edge, positioning of the hands on the table and time to perform the stitches, described in models used in the development of basic skills, such as tomatoes^2^, grapes^13^, and green beans^24^, to the development of advanced skills^25^ ^-^ ^27^.

Since the objective of the research was solely the description of the development of a microsurgical training model with corn grains, the study lacks specific criteria for validating the simulator, however the established model is worthy of future legitimization scientific protocols in gaining microsurgical skills.

## CONCLUSION

The training model developed from corn grains was shown to be viable for training basic manual skills in microsurgery. In addition, the proposed simulator has low cost and good availability. Therefore, the model can be readily adapted to the practice of microsurgical suturing by undergraduates or residents. Since this study lacks specific validation protocols, the simulator still has future research opportunities for its use to be accredited and systematized in the microsurgical learning process.
